# Amniotic fluid antiphospholipid antibodies: potential role in antiphospholipid syndrome-independent aberrant implantation process

**DOI:** 10.1186/s12958-019-0527-2

**Published:** 2019-10-15

**Authors:** Valentina Bruno, Marzia Nuccetelli, Carlo Ticconi, Antonella Bruno, Federica Martelli, Maria Vittoria Capogna, Sergio Bernardini, Emilio Piccione, Adalgisa Pietropolli

**Affiliations:** 1 Academic Department of Biomedicine and Prevention, Section of Gynecology, University of Rome “Tor Vergata”, Rome, Italy; 2 Department of Experimental Medicine and Surgery, Tor Vergata University Hospital, Tor Vergata University, Rome, Italy; 3 Academic Department of Surgical Sciences, Section of Gynecology, Tor Vergata University Hospital, University of Rome “Tor Vergata”, 00133 Rome, Italy; 4 Academic Department of Systems Medicine, University of Rome “Tor Vergata”, Rome, Italy; 5 Department of Surgical Sciences, Section of Gynecology, Tor Vergata University Hospital, Rome, Italy

**Keywords:** Antiphospholipid antibodies, Amniotic fluid, Impaired implantation, Pregnancy complications

## Abstract

**Background:**

The direct role of antiphospholipid antibodies (aPL) at maternal-fetal interface has not been fully investigated, especially whether they are involved in physiological and pathological implantation conditions, in an antiphospholipid syndrome (APS)-independent manner. In fact, trophoblast cells and placental endothelial cells at the implantation site express potential aPL targeted-phospholipid antigens (PL Ags); thus, the local production and presence of their specific antibodies, not related to APS (characterized by aPL presence in the peripheral blood), could be a potential marker of aberrant invasion, implantation and fetal-maternal immune tolerance processes.

**Methods:**

Anti-Beta_2_glycoprotein I (anti-β_2_GPI) and anticardiolipin (aCL Ab) antibodies (the most clinically relevant aPL) were detected by immunoenzymatic assay (ELISA), in the amniotic fluid (AF) of 167 women with physiological and complicated common pregnancy conditions, sharing an aberrant implantation process, such as recurrent pregnancy loss (RPL), autoimmune hypothyroidism (ahT) and smoking. All women included in the study were negative to peripheral blood aPL.

**Results:**

aCL and anti-β_2_GPI antibodies were detectable in all the AF samples. RPL, ahT and smoking patients had higher level of anti-β_2_GPI Abs (IgM) compared to women with physiological pregnancies (*p* < 0.0001). Since IgM cannot cross the placenta, their local production in response to maternal-fetal interface *stimuli,* could be hypothesized.

**Conclusions:**

The presence of aPL in the AF (not related to APS) could reveal a potential clinical significance at maternal-fetal interface in selected pregnancy complications, in which an aberrant implantation process, and in turn an impaired fetal-maternal immune tolerance cross-talk, could occur.

## Introduction

Antiphospholipid antibodies (aPL) are a heterogeneous group of autoantibodies, whose systemic expression is related to the antiphospholipid syndrome (APS) [1]. APS is clinically characterized by thrombotic events and pregnancy complications, such as recurrent pregnancy loss (RPL), preeclampsia, fetal growth restriction, infertility. The diagnostically relevant-aPL, necessary for APS diagnosis together with the clinical manifestations are: LAC (lupus anti-coagulant activity), anti-β_2_GPI and aCL Abs [2, 3].

Human β_2_-glycoprotein I (β_2_GPI) is the major antigen recognized as a target for antiphospholipid antibodies [4, 5] and has been showed to be expressed by placental endothelial cells and placenta itself [6]. Among antiphospholipid antibodies, those specific for β2GPI are considered as the most clinically relevant for in pregnancy complications [7, 8]. β2GPI is expressed in human extravillous trophoblast cell membrane, thus making these cells a target for aPL [9–11].

By binding trophoblast-expressed β2GPI, their specific antibodies, caused dysfunctional modifications, such as a reduced human chorionic gonadotropin secretion and trophoblast invasiveness [12, 13], responsible for a defective placentation process.

In fact, it has been demonstrated that APS-induced complications in pregnancy are caused by direct effects of the aPL on trophoblasts, leading to an impaired trophoblast invasion [9, 14, 15]. This mechanism is confirmed by the abnormal expression of integrins and MMPs profiles (essential for feto-maternal interface and invasion process functionality), which aPL are responsible for [16–18], with a particular regard in vitro for monoclonal antibody (MAb) against β_2_GPI [19].

Furthermore, anti-β_2_GPI MAb could have a potential role in disrupting feto-maternal tolerance process during invasion and implantation, by affecting galectin-1, an important immunomodulatory protein involved in regulatory T cells generation and recruitment at the implantation site, in order to avoid embryo rejection [20].

Moreover, anticardiolipin antibodies (aCL) are involved in adverse pregnancy outcomes, since they can cause decidual vasculopathy, uteroplacental insufficiency, placental thrombosis and infarction. The presence of aCL IgG in the amniotic fluid has been demonstrated in patients affected by APS, but not in a control group women [21]. There is no information, instead, about the presence of anti-β_2_GPI Ab in the amniotic fluid.

The aim of the study is to investigate the presence of the two most specific aPL in amniotic fluid, aCL and anti-β_2_GPI antibodies, in case of their absence in peripheral blood, to determine if they have a potential role in physiological and pathological pregnancy implantation processes, not related to APS. For this purpose amniotic fluid aPL were measured in women with physiological pregnancy and pregnant women affected by unexplained RPL, autoimmune hypothyroidism - a common autoimmune disease which has a role in pregnancy outcome - and in smoking pregnant women. These conditions were chosen since they are common in general population and share similar pathways related to a not proper implantation process, and thus they can be a challenging model to investigate local aPL dowstreaming effects. The scientific rationale beyond, rises from the knowledge that phospholipid antigens are located in the throphoblast cell membrane and in placental endothelial cells at the implantation site, and thus the presence of their related antibodies could be a potential marker of aberrant invasion, implantation and feto-maternal immune tolerance processes. To summarize, our research question was to investigate the potential direct role of the influence of aPL in these pathological conditions in pregnancy, not related to the clinical manifestation of the APS, since its effect on pregnancy outcome is already well known: for that purpose we enrolled only patients with an unexplained RPL.

## Materials and methods

### Subjects and procedures

This prospective study involved 167 caucasian women of reproductive age. Subjects were divided into four groups:

1. Forty-seven no-smoking healthy women with current physiological pregnancy, at least 2 previous at term pregnancies and without any miscarriages or autoimmune disease (control group).
2. Thirty-six no-smoking healthy women affected by uRPL (defined as 2 or more consecutive abortions before 24th week of gestation, according to the ESHRE guidelines 2017) [22], without any autoimmune disease (RPL group).
3. Forty smoking healthy women with current physiological pregnancy, at least 2 previous at term pregnancies and without any miscarriages or autoimmune disease (smoking group).
4. Forty-four no-smoking healthy women with current physiological pregnancy, at least 2 previous at term pregnancies and without any miscarriages affected by autoimmune hypothyroidism (ahT- diagnosis made by elevated TPOAb and/or TgAb above 350 IU/ml and basal TSH) [23], but not by other autoimmune diseases (ahT group).

All the women included in this study attended as outpatients the Complex Operative Unit of Gynecology and Obstetrics of the University Hospital Policlinico Tor Vergata, Rome. Women with RPL were followed at the RPL Unit, whereas controls were followed at the Physiological Pregnancy Outpatients Clinic.

Patients were recruited at the amniocentesis stage, and then retrospectively excluded from previous RPL evaluations. Indeed, these patients were previously followed by our RPL unit, before pregnancy, and underwent our standardized diagnostic work-up, which included the following: a) collection of familial and personal medical, gynecological and obstetrical history with specific application to the previous miscarriages; b) gynecological examination; c) transvaginal ultrasound; d) hysteroscopy and, when appropriate, endometrial biopsy; e) endocrine evaluation panel: assay of LH, FSH, prolactin, progesterone in the midluteal phase, TSH, FT3, FT4, pituitary and ovarian androgens, insulin and glucose curve; e) karyotype of both partners; f) immunity panel: lupus anticoagulant, anti-β_2_GPI, aCL and anti-annexin V antibodies, anti-thyroid antibodies, *antinuclear* antibody (ANA), extractable nuclear antigen (ENA), anti-double stranded DNA antibody (anti-ds DNA), anti-smooth muscle antibody (ASMA) and anti-mitochondrial antibody (AMA); g) thrombophilia screening: protein C, protein S, AT III, APCR, homocysteine; determination of the following mutations: factor V [G1691A Leiden], factor II prothrombin [G20210 A], plasminogen activator inhibitor [PAI-1 4G/5G], methylen tetrahydrofolate reductase [MTHFR C677T and A1298C]. This workup was aimed to identify proven, probable and doubtful causes of RPL [24–27].

When all the above known causes for pregnancy loss had been discarded, women were diagnosed with unexplained RPL and were included in this study. Women with a history of at least 2 normal pregnancies at term, without any miscarriage were followed according the standardized protocol used in our unit, which complies with the NICE Clinical Guidelines [28].

In a first step, all women underwent amniocentesis, because of a 35 or more years old maternal age, positive first trimester screening, parental genetic disease, previous offspring affected by chromosomal abnormalities, were included in the study. In a second step, only patients received amniocentesis due to maternal age, were included in data analysis. Patients with other indications, such as positive first trimester screen or parental genetic diseases, were excluded and therefore not included in the analysis, since these factors may be considered as a bias, and therefore they would have affected the outcome variable.

Only singleton pregnancies were included in the study, and amniocentesis was performed at a gestational age of 16–18 weeks. Pregnancies with a fetus affected by chromosomal defects were excluded from this study.

An ultrasound check has been performed for all patients before amniocentesis by using an ultrasound Hitachi Logos equipped with a transabdominal 3,5 MHz probe. Fetal morphology, placental insertion, amniotic fluid, and FHR were checked. The skin was sterilized by topical povidone-iodine solution application. Amniocentesis was performed in a sterile environment and under continuous ultrasound guidance, by using a 20-gauge 20-cm needle. The easiest accessible and deepest amniotic fluid pocket, which ensure the minimal fetal presence was chosen, taken also into account maternal viscera and vasculature safety. The first milliliter of amniotic fluid was discharged as a standard protocol in clinical practice, and other 20 ml were collected for the cell culture. An ultrasound check was performed soon after the procedure and one hour after the procedure [29].

After the amniocentesis, the discarded aliquot of amniotic fluid, after fetal karyotyping, was used to assess the presence of aCL antibodies IgM and IgG, and anti-β_2_GPI antibodies IgM and IgG. These discarded aliquots were obtained only after written informed consent. The present study was carried out in accordance with the Declaration of Helsinki, modified Tokyo 2004, and was approved by the Institutional Review Board (IRB) of Policlinico Tor Vergata University Hospital (protocol number: R.S. 64/15). Any collected information was anonymised and de-identified prior to analysis.

### aPL and anti-β_2_GPI assessment

Anti-cardiolipin antibodies (IgG/IgM) and anti-beta2 glycoprotein I antibodies (IgG/IgM) were detected using commercial ELISA kits by INOVA Diagnostics (San Diego, CA, USA). Two hundred microliters of patient amniotic fluid (undiluted) and standard calibrators (ranging from 0 to 100 phospholipid units – MPL, GPL) were dispensed in duplicate and incubated for 30 min. After incubation with HRP (Horse Radish Peroxidase) IgG or IgM conjugate (30 min), the reaction was detected with the addition of a substrate solution (TMB chromogen). The absorbance of the colorimetric reaction was read at 450 nm. The sample concentrations were calculated on the corresponding standard curve equation.

### Statistical analysis

Kolmogorov-Smirnov test was used to analyze data distribution. Data are expressed as mean ± standard and median (range), as appropriated. Women’s clinical characteristics have been analyzed by one way ANOVA followed by Tukey’s multiple comparisons test. Statistical analysis has been carried out by using student’s t test, since data were normally distributed. Significance was set at *p* ≤ 0.05. Statistical analyses were performed using GraphPad Prism ver. 7.0 (GraphPad Software, San Diego, CA, USA).

## Results

### Clinical characteristics of study women

Clinical characteristics of women enrolled in the study are reported in Table 1. No significant differences were detected in patients’ age and body mass index (BMI). All unexplained RPL patients were negative to the standardized diagnostic work-up, previously described. All groups were negative to peripheral blood APS assays.

**Table 1:** Clinical characteristics in study women

	RPL Group (*n* = 36)	Smoke Group (*n* = 40)	ahT Group (*n* = 44)	Control Group (*n* = 47)
Maternal age	39,3 ± 1,4	37,5 ± 3,3	36,2 ± 1,2	38,4 ± 3,4
BMI (Kg/m^2^)	23 ± 2,7	24,7 ± 4,6	26,2 ± 6,3	23,9 ± 4,6
Parity	0,5 (0–2)	2 (2–3)	2 (2–3)	2 (2–3)
*N. of miscarriages*	2,6+ 1,3	–	–	–
Gestational week of miscarriage	8,7+ 1,4	–	–	–

Data are expressed as Mean + S.D. or median and range, as appropriate

*RPL* recurrent pregnancy loss

*ahT* autoimmune hypothyroidism

### aCL and anti-β_2_GPI antibodies (abs) assays

aCL (IgM, IgG) and anti-β_2_GPI (IgM, IgG) antibodies were found at detectable concentrations in all the amniotic fluid samples.

**Fig. 1:**
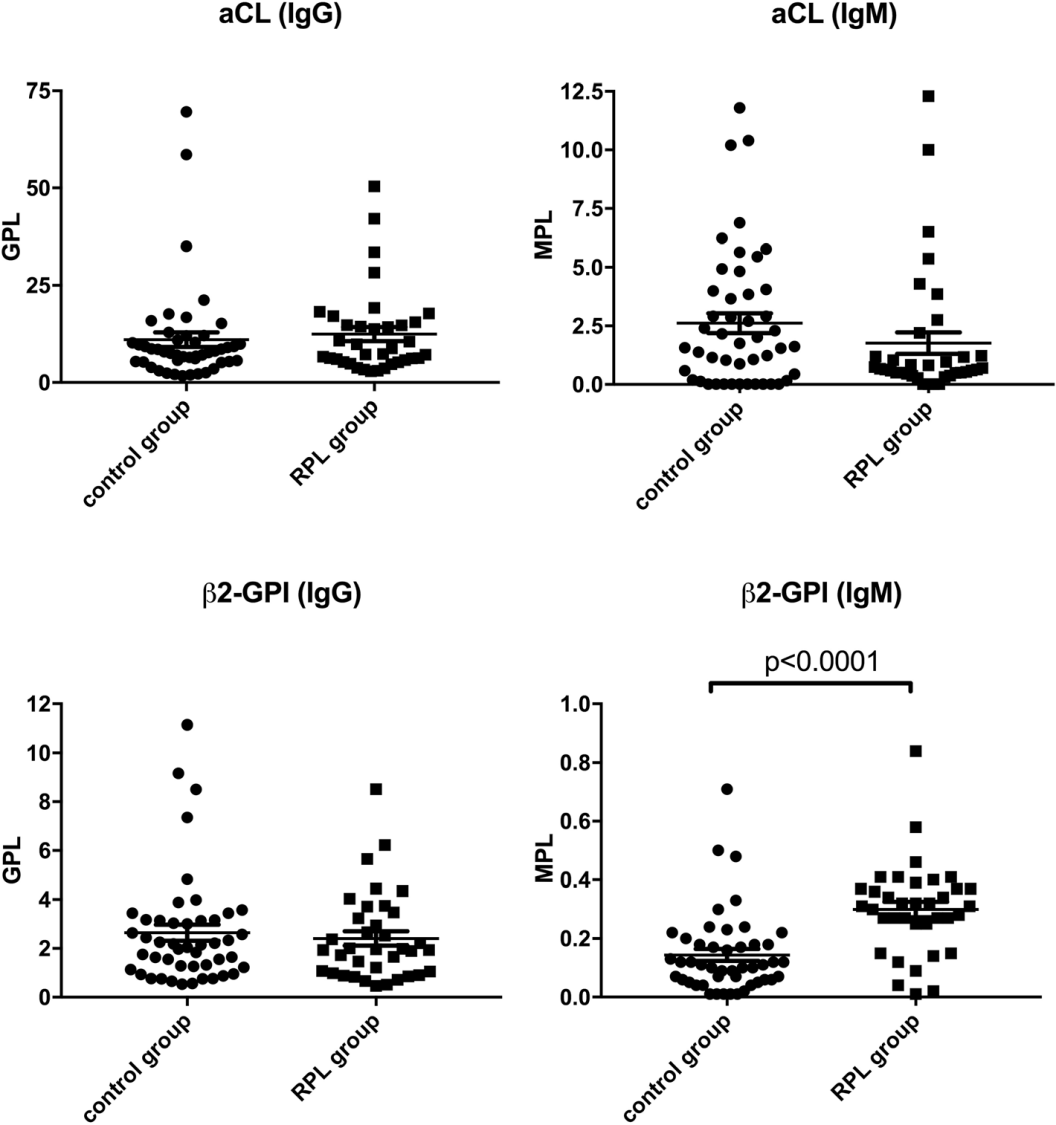
aCL (IgM, IgG) and anti-β_2_GPI (IgM, IgG) Abs levels (MPL and GPL, respectively) in control group vs RPL group. *Student’ s t test: *p* < 0,0001

**Fig. 2:**
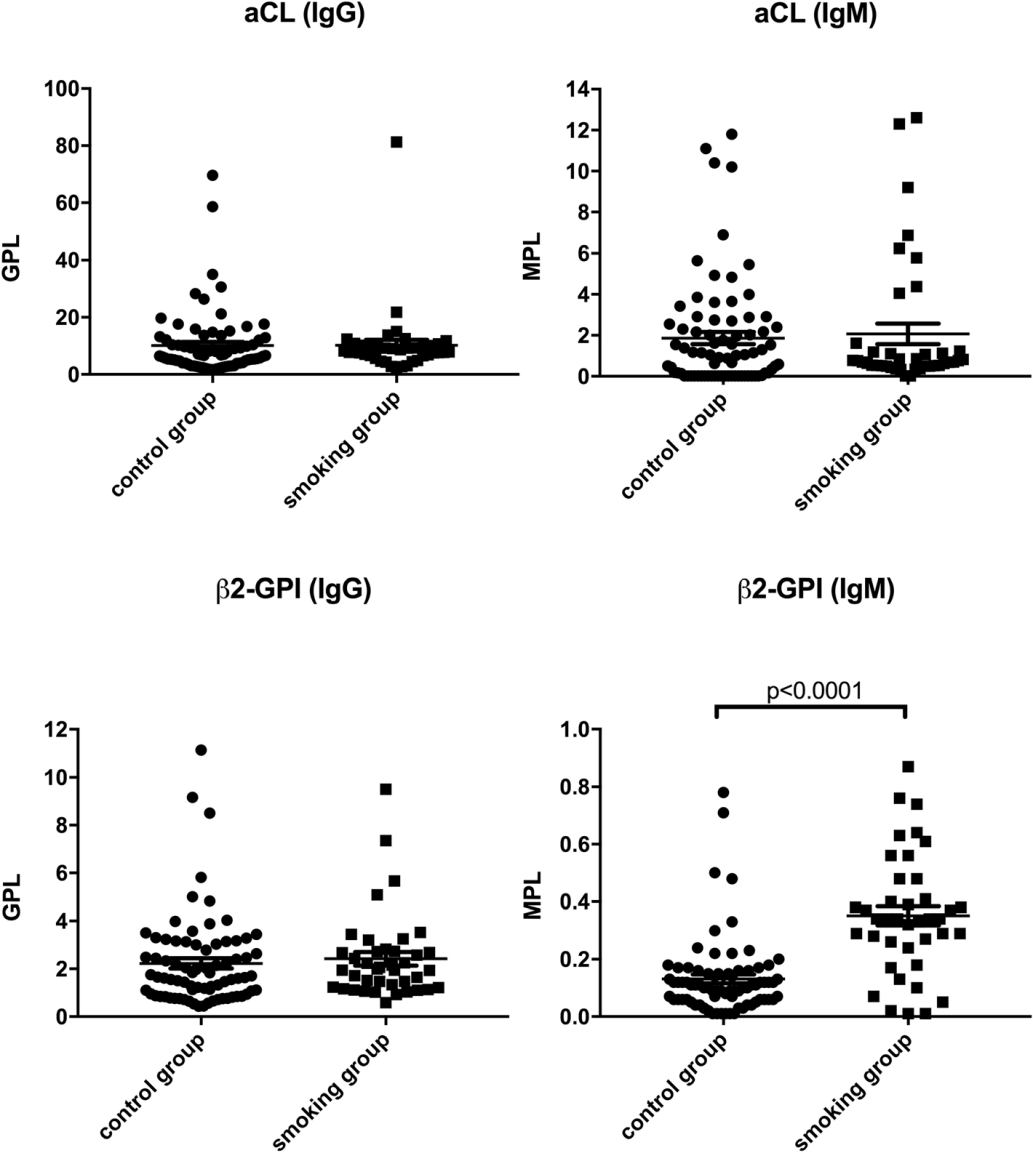
aCL (IgM, IgG) and anti-β_2_GPI (IgM, IgG) Abs levels (MPL and GPL, respectively) in control group vs smoking group. *Student’ s t test: *p* < 0,0001

**Fig. 3:**
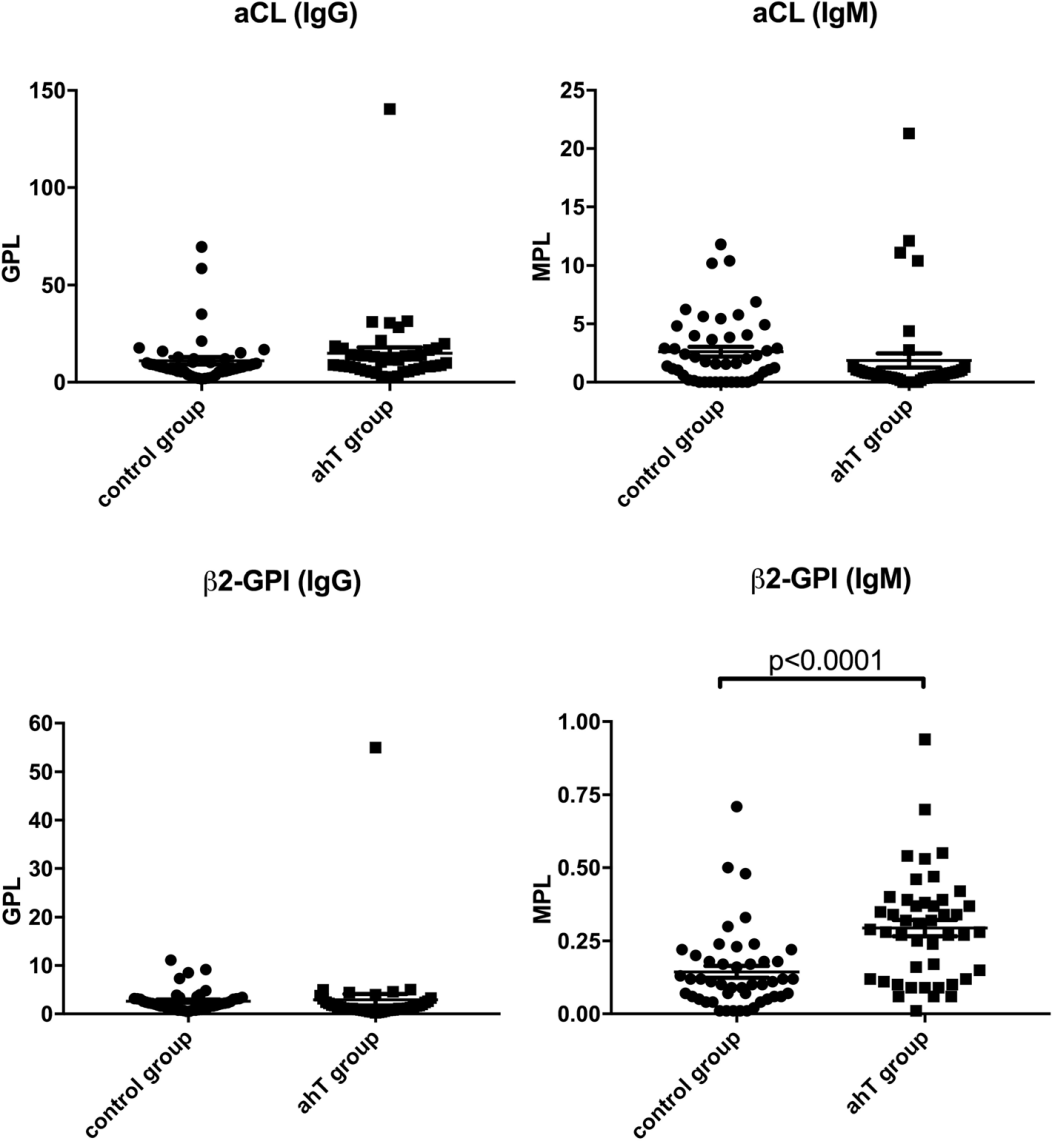
aCL (IgM, IgG) and anti-β_2_GPI (IgM, IgG) Abs levels (MPL and GPL, respectively) in control group vs ahT group. *Student’ s t test: *p* < 0,0001

Higher level of anti-β_2_GPI Abs (IgM) (*p* ≤ 0.0001) were detected in the amniotic fluid (AF) of pregnant women belonging to RPL group, smoking group and autoimmune hypothyroidism (ahT) group compared with women with physiological pregnancies (control group) (Figs. 1, 2 and 3). Anti-cardiolipin (IgM, IgG) and anti-β_2_GPI Abs (IgG) levels were also investigated, however no differences were found among these groups (Figs. 1, 2 and 3).

## Discussion

The main finding pointed out by our investigation is that all autoantibodies assayed in the amniotic fluid were observed to be detectable in all patients. At the best of our knowledge this is the first study in which aCL and anti-β_2_GPI Abs have been detected in the amniotic fluid of patients negative to the peripheral blood tests for aPL Abs.

These data could have a potential clinical implication suggesting a physiological role of the local production of these antibodies in pregnancy and a possible role of their level modifications in selected complications of pregnancy.

Another major finding of the present study is that women with RPL, autoimmune hypothyroidism or smoking behavior have an important statistically significant difference in anti-β_2_GPI Abs (IgM) levels compared to control group. The higher levels in these pathological conditions were detected only for IgM class, supporting the idea of a local production of the investigated Abs, since all patients were negative for aPL Abs tests in peripheral blood and IgM cannot cross the placental barrier. Furthermore, fetus at the gestational weeks in which amniocentesis is performed (mostly at 16 weeks of gestation and anyway between 16 and 18 weeks of gestation), do not produce himself IgM (since his IgM production starts at 20 weeks of gestation) [30]. By putting together all the above considerations, we can assume that IgM anti-β_2_GPI Abs could come from the placenta or at least from the immunological infiltrate at the fetal-maternal interface; to further support this hypothesis an endogenous production of antibodies has been demonstrated: cultured trophoblast cells have been shown to produce IgG and transcripts encoding IgG molecules have been detected in this cell line [31].

Scientific literature strengthens this hypothesis, underlining the β_2_GPI presence only as a phospholipid antigen in trophoblasts cell membrane and placental endothelial cells, especially at the implantation site, as well as in the liver [6]. The pathological conditions taken into account are characterized by an aberrant implantation process, caused by anomalies in the immunological (RPL and ahT) and oxidative stress (RPL and smoking) physiological balances, leading to placental endothelial dysfunction, dysregulation in neo-angiogenesis pathways and accelerated apoptotic processes in trophoblast cell line [24, 32–35]. The presence of anti-β_2_GPI Abs in the AF could represent a direct marker of placental dysfunction and of abnormal implantation, since an accelerated apoptotic process could expose the β_2_GPI antigen from trophoblast cells disrupted membrane. A potential explanation about the detection of these antibodies in physiological pregnancy could be based on the continuous remodeling at the maternal-fetal interface in order to ensure adequate modifications to the developing placenta and fetus, during different stages of pregnancy. However, since these elevated auto-antibodies could also be only a response to some pathological conditions (such as autoimmune hypothyroidism, RPL unrelated to APS or smoking), but might not actually be pathological, further studies are needed to understand the pathophysiological local mechanism at the maternal-fetal interface related to the presence of aPL Abs in the amniotic fluid. Based on these observed results, and due to the low number of patients followed thorough all pregnancy steps by our team, and in turn their few known adverse obstetrical outcomes available in our medical records, the next step would be the realization of a randomized trial on a bigger cohort, to investigate from a clinical point of view, the potential in vivo effects of these laboratory findings.

## Conclusions

Based on our findings, antiphospholipid antibodies, and especially anti-β_2_GPI Abs (IgM), could have an APS- independent role in physiological pregnancy and in selected pregnancy complications. In particular IgM anti-β_2_GPI Abs could be a potential marker of an aberrant implantation process, with a consequence on the developing fetal-maternal immune tolerance cross-talk.

## Data Availability

The datasets used and analysed during the current study are available from the corresponding author on reasonable request.

## References

[1] Hughes GRV, Khamashta MA (1994). The antiphospholipid syndrome. J R Coll Physicians Lond.

[2] Miyakis S, Lockshin MD, Atsumi T (2006). International consensus statement on an update of the classification criteria for definite antiphospholipid syndrome (APS). J Thromb Haemost.

[3] Wilson WA, Gharavi AE, Koike T, Lockshin MD (1999). International consensus statement on preliminary classification criteria for definite antiphospholipid syndrome: report of an international workshop. Arthritis Rheum.

[4] Fischetti F, Durigutto P, Pellis V (2005). Thrombus formation induced by antibodies to beta2-glycoprotein I is complement dependent and requires a priming factor. Blood.

[5] Pierangeli SS, Vega-Ostertag ME, Raschi E (2007). Toll-like receptor and antiphospholipid mediated thrombosis: *in vivo* studies. Ann Rheum Dis.

[6] Agostinis C, Biffi S, Garrovo C, Durigutto P, Lorenzon A, Bek A, Bulla R, Grossi C, Borghi MO, Meroni PL, Tedesco F (2011). *In vivo* distribution of b2 glycoprotein I under various pathophysiologic conditions. BLOOD.

[7] Meroni PL, Borghi OM, Raschi E, Tedesco F (2011). Pathogenesis of antiphospholipid syndrome: understanding the antibodies. Nat Rev Rheumatol.

[8] Robertson SA, Roberts CT, van Beijering E (2004). Effect of beta2-glycoprotein I null mutation on reproductive outcome and antiphospholipid antibody-mediated pregnancy pathology in mice. Mol Hum Reprod.

[9] Di Simone N, Meroni PL, Del Papa N (2000). Antiphospholipid antibodies affect trophoblast gonadotropin secretion and invasiveness by binding directly and through adhered beta2-glycoprotein I. Arthritis Rheum.

[10] Chamley LW, Allen JL, Johnson PM (1997). Synthesis of beta2 glycoprotein 1 by the human placenta. Placenta.

[11] Agostinis C, Biffi S, Garrovo C (2011). *In vivo* distribution of b2 glycoprotein I under various pathophysiological conditions. Blood.

[12] Di Simone N, Meroni PL, D’Asta M, Di Nicuolo F, D’Alessio MC, Caruso A (2007). Pathogenic role of antibeta2- glycoprotein I antibodies on human placenta:functional effects related to implantation and roles of heparin. Hum Reprod Update.

[13] Meroni PL, Tedesco F, Locati M (2010). Anti-phospholipid antibody mediated fetal loss: still an open question from a pathogenic point of view. Lupus.

[14] Di Simone N, Caliandro D, Castellani R, Ferrazzani S, De Carolis S, Caruso A (1999). Low molecular weight heparin restores in vitro trophoblast invasiveness and differentiation in presence of immunoglobulin G fractions obtained from patients with antiphospholipid syndrome. Hum Reprod.

[15] Jovanović M, Bozić M, Kovacević T, Radojcić L, Petronijević M, Vićovac L (2010). Effects of anti-phospholipid antibodies on a human trophoblast cell line (HTR-8/ SVneo). Acta Histochem.

[16] Di Simone N, Castellani R, Caliandro D, Caruso A (2002). Antiphospholipid antibodies regulate the expression of trophoblast cell adhesion molecules. Fertil Steril.

[17] Staun-Ram E, Shlomit G, Gabarin D, Shalev E (2004). Expression and importance of matrix metalloproteinase 2 and 9 (MMP-2 and -9) in human trophoblast invasion. Reprod Biol Endocrinol.

[18] Di Simone N, Marana R, Castellani R (2010). Decreased expression of heparin binding epidermal growth factor-like growth factor as a newly identified pathogenic mechanism of antiphospholipid-mediated defective placentation. Arthritis Rheum.

[19] Kovačević TM, Radojčić L, Tošić NM, Pavlović ST, Vićovac LM (2013). Monoclonal antibody 26 cross-reactive with b2-glycoprotein I affects human trophoblast invasion in vitro. Eur J Obstet Gynecol Reprod Biol.

[20] Blois SM, Ilarregui JM, Tometten M (2007). A pivotal role for galectin-1 in fetomaternal tolerance. Nat Med.

[21] Cohen SB, Goldenberg M, Rabinovici J, Lidor AL, Dulitzky M, Gilburd B, Shoenfeld Y, Schiff E (2000). Anti-cardiolipin antibodies in fetal blood and amniotic fluid derived from patients with the anti-phospholipid syndrome. Hum Reprod.

[22] RECURRENT PREGNANCY LOSS (2017). Guideline of the European society of human reproduction and embryology. ESHRE Early Pregnancy Guideline Development Group. NOVEMBER.

[23] Gärtner R, Gasnier BC, Dietrich JW, Krebs B, Angstwurm MW (2002). Selenium supplementation in patients with autoimmune thyroiditis decreases thyroid peroxidase antibodies concentrations. J Clin Endocrinol Metab.

[24] Christiansen OB, Steffenson R, Nielsen H, Varming K (2008). Multifactorial etiology of recurrent miscarriage and its scientific and clinical implications. Gynecol Obstet Invest.

[25] Pietropolli A, Giuliani E, Bruno V, Patrizi L, Piccione E, Ticconi C (2014). Plasminogen activator inhibitor-1, factor V, factor II and methylenetetrahydrofolate reductase polymorphisms in women with recurrent miscarriage. J Obstet Gynaecol.

[26] Pietropolli A, Bruno V, Capogna MV, Bernardini S, Piccione E, Ticconi C (2015). Uterine blood flow indices, antinuclear autoantibodies and unexplained recurrent miscarriage. Obstet Gynecol Sci.

[27] Ticconi C, Pietropolli A, Borelli B, Bruno V, Piccione E, Bernardini S, Di Simone N (2016). Antinuclear autoantibodies and pregnancy outcome in women with unexplained recurrent miscarriage. Am J Reprod Immunol.

[28] National Institute for Health and Clinical Excellence (NICE) (2008). Antenatal care for uncomplicated pregnancies. Clinical Guideline CG62.

[29] Pietropolli A, Martelli F, Vicario R, Montagnoli C, Ticconi C, Piccione E (2011). Evaluation of fetal heart rate variation during amniocentesis: correlation with fetal karyotype. J Matern Fetal Neonatal Med.

[30] van Furth R, Schuit HR, Hijmans W (1965). The immunological development of the human fetus. J Exp Med.

[31] Gu J, Lei Y, Huang Y, Zhao Y, Li J, Huang T, Zhang J, Wang J, Deng X, Chen Z, Korteweg C, Deng R, Yan M, Xu Q, Dong S, Cai M, Luo L, Huang G, Wang Y, Li Q, Lin C, Su M, Yang C, Zhuang Z (2015). Fab fragment glycosylated IgG may play a central role in placental immune evasion. Hum Reprod.

[32] Matthiesen L, Kalkunte S, Sharma S (2012). Multiple pregnancy failures: an immunological paradigm. Am J Reprod Immunol.

[33] Ticconi C, Giuliani E, Veglia M, Pietropolli A, Piccione E, Di Simone N (2011). Thyroid autoimmunity and recurrent miscarriage. Am J Reprod Immunol.

[34] Valentina B, Barbara R, Adalgisa P, Vittoria CM, Renato M, Carlo T, Emilio P, Claudio C, Giuseppe N, Francesca A (2017). OLR1 and Loxin expression in PBMCs of women with a history of unexplained recurrent miscarriage: a pilot study. Genet Test Mol Biomarkers.

[35] Dechanet C, Brunet C, Anahory T, Hamamah S, Hedon B, Dechaud H (2011). Effects of cigarette smoking on embryo implantation and placentation and analysis of factors interfering with cigarette smoke effects (part II). Gynecol Obstet Fertil.

